# Analysis and comparison of tear protein profiles in dogs using different tear collection methods

**DOI:** 10.1186/s12917-022-03543-7

**Published:** 2022-12-21

**Authors:** Sudpatchara Ritchoo, Phattara-orn Havanapan, Nuanwan Phungthanom, Rucksak Rucksaken, Rattana Muikaew, Metita Sussadee

**Affiliations:** 1 Department of Veterinary Technology, Faculty of Veterinary Technology, Kasetsart University, Bangkok, Thailand; 2 Institute of Molecular Biosciences, Mahidol University, Salaya Campus, Nakhonpathom, Thailand

**Keywords:** Tear, Protein profile, Schirmer tear strip, Ophthalmic sponge, Microcapillary tube, Two-dimensional electrophoresis, Dog

## Abstract

**Background:**

Tear proteomic analysis has become an important tool in medical and veterinary research. The tear collection method could influence the tear protein profile. This study aims to evaluate the protein profiles of dog tears collected using microcapillary tubes (MT), Schirmer tear strips (ST), and ophthalmic sponges (OS).

**Methods:**

The tear samples were collected using MT, ST, and OS. Tear protein profiles were analyzed using two-dimensional electrophoresis (2-DE) and the different protein spots’ expression was compared. Fourteen protein spots were identified using liquid chromatography-tandem mass spectrometry (LC-MS/MS).

**Results:**

Tear protein concentrations ranged from 2.80 to 4.03 μg/μL, with no statistically significant differences among collection methods. Protein expression in each collection method differed in terms of both the number and intensity of the spots. There were 249, 327, and 330 protein spots found from tears collected with MT, ST, and OS, respectively. The proteins albumin, haptoglobin, and lactoferrin identified from OS were found to have higher spot intensities than other methods of collection. The use of MT demonstrated the downregulation of nine proteins.

**Conclusions:**

The recent study supported that tear protein analysis is affected by different tear collection methods. Although ST is commonly used for tear collection, it provides insufficient information to study particular tear proteins.

**Supplementary Information:**

The online version contains supplementary material available at 10.1186/s12917-022-03543-7.

## Background

Tear film, a thin liquid layer that completely covers the ocular surface, is the first barrier between the outer environment and the eyes. The main functions include moistening and lubricating the ocular surface, providing oxygen and electrolytes to the cornea, improving the refractive power of the eye, and protecting the eye from a potentially damaging environment [1]. Tears are generally classified into three layers. The outer lipid is crucial for the film’s stability and prevents the underlying aqueous layer from rapidly evaporating. The middle aqueous layer contains electrolytes, proteins, and small-molecule metabolites. The inner mucin layer interacts with the corneal epithelial surface. Tear mucus composed of water and mucin glycoproteins serves to maintain barrier function and wettability of the hydrophobic surface epithelial cell membranes, provide a matrix for lacrimal secreted factors, and minimize friction from blinking [2–4]. Many biologically active molecules have been found in tears [5]. The protein composition of the tear film can be altered by various local and systemic diseases [1]. Tear protein analysis can facilitate the identification of new biomarkers, a better understanding of disease etiology, and the development of new diagnostic tools and therapies [6]. In addition to its clinical utility, the identification of biomarkers in tear film may be useful in developing new pharmacologically active molecules [7]. Nowadays, tear protein analysis is used for diagnosis and following disease status in the medical and veterinary fields. In humans, tear protein biomarkers are used to determine dry eye syndrome [8], glaucoma [9], and Parkinson’s disease [10]. In veterinary medicine, tear protein biomarkers are used to determine dry eye syndrome [11], and various cancers including transmissible venereal tumor, mammary gland adenocarcinoma, and skin malignant melanoma [12].

For the tear protein analysis process, the most appropriate tear collection method is crucial in tear protein study. There are two tear sampling methods performed on animals, including direct sampling (microcapillary glass tubes) and indirect sampling (Schirmer tear strips and ophthalmic sponges) [6, 13–15]. It is evident that the various methods have different advantages and limitations [16]. Moreover, it is unclear what the ideal method of tear collection is for minimizing protein degradation and providing the important information for proteomic study. Schirmer’s tear test-1 or ST is the routine method for clinical ocular examination and dry eye diagnosis in dogs [3, 17]. ST is performed by inserting the folded end of the strip into the lower conjunctival fornix for 1 minute without the use of topical anesthetics [18]. Normal STT-1 values in dogs range from 15 to 25 mm/min [19]. In comparison to the other methods, ST has a lower coefficient of variability in tear protein content. It can provide the reliability and reproducibility of the data [16]. Despite the fact that ST has been considered convenient and easy, it can cause strong irritation of the conjunctiva, and trigger reflex tearing [20, 21]. The MT is the most commonly reported technique to collect tear film. This method is extensively described in humans, dogs, rabbits, mice, and rats. MT can be performed by placing a blunt capillary tube at the corner of the eye, within the tear lake, for around 5 minutes [1]. It is possible to obtain unaltered tear samples by avoiding reflex tearing from ocular irritation. Thus, the tear quality is similar to the basal tear. However, it takes a long time, produces a small volume, and is difficult to collect [6, 16]. The OS is available in different material types such as cellulose, polyvinyl acetal (PVA), polyester, and polyurethane [16]. For tear collection, OS is held against the lacrimal gland or placed beneath the lower eyelid for a given period of time [16]. The OS provides rapid absorbance and has a high absorptive capacity. Consequently, OS is suitable for further use as a blank tear in bioanalytical assays. However, this method is invasive, can promote reflex tearing, and might alter the composition of the tear film [15, 16].

Previous studies have shown differences in protein expression when using various protein analysis and collection methods [13, 17, 22], but no studies have investigated the difference in expression of healthy dog tears using 2-DE gel electrophoresis. The primary objective of this study was to evaluate the protein profiles of dog tears collected using three different tear collection methods: MT, ST, and OS.

## Results

**Fig. 1:**
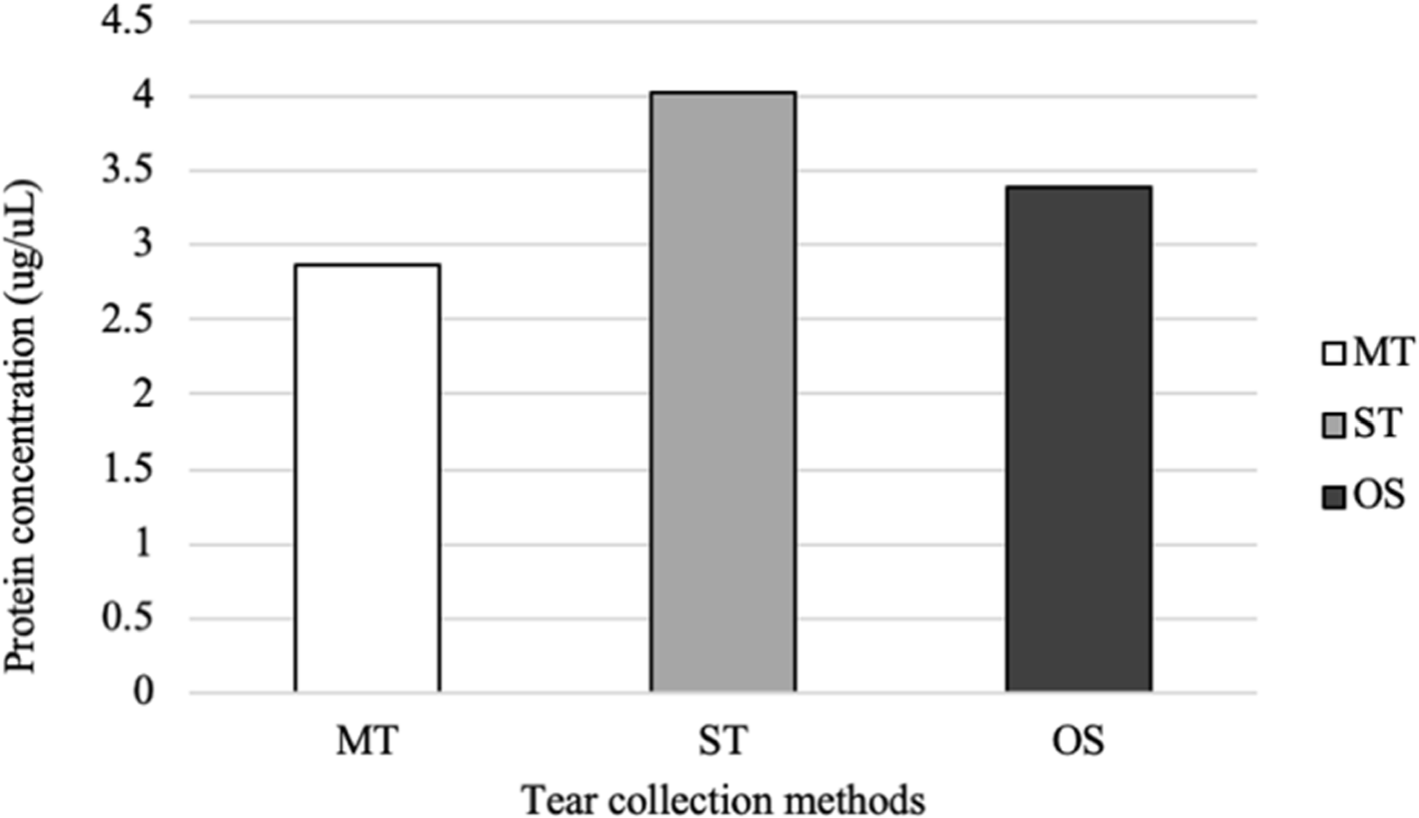
Total protein concentration in canine tears, as determined using the Bradford assay. Tear samples were collected with MT, ST, and OS

The mean (± SD) protein concentrations in tears collected with MT, ST, and OS were 2.87 ± 0.62, 4.03 ± 1.17, and 3.39 ± 0.69 μg/μL, respectively. There were no statistically significant differences found between the tear collection methods (*P- value* = 0.22) (Fig. 1).

**Fig. 2:**
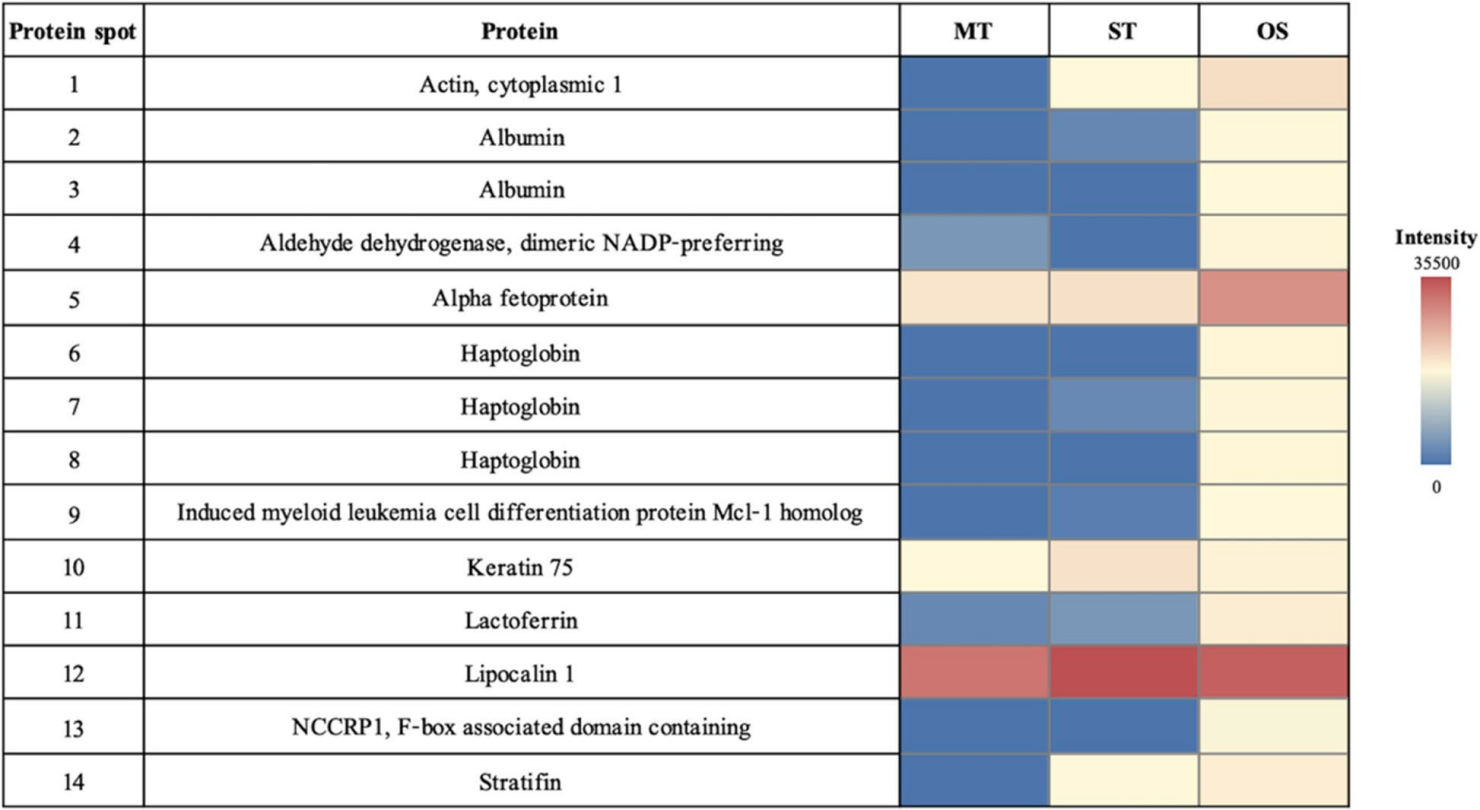
Heatmap showing the relative abundance (color) of 14 candidate proteins in MT, ST, and OS

**Fig. 3:**
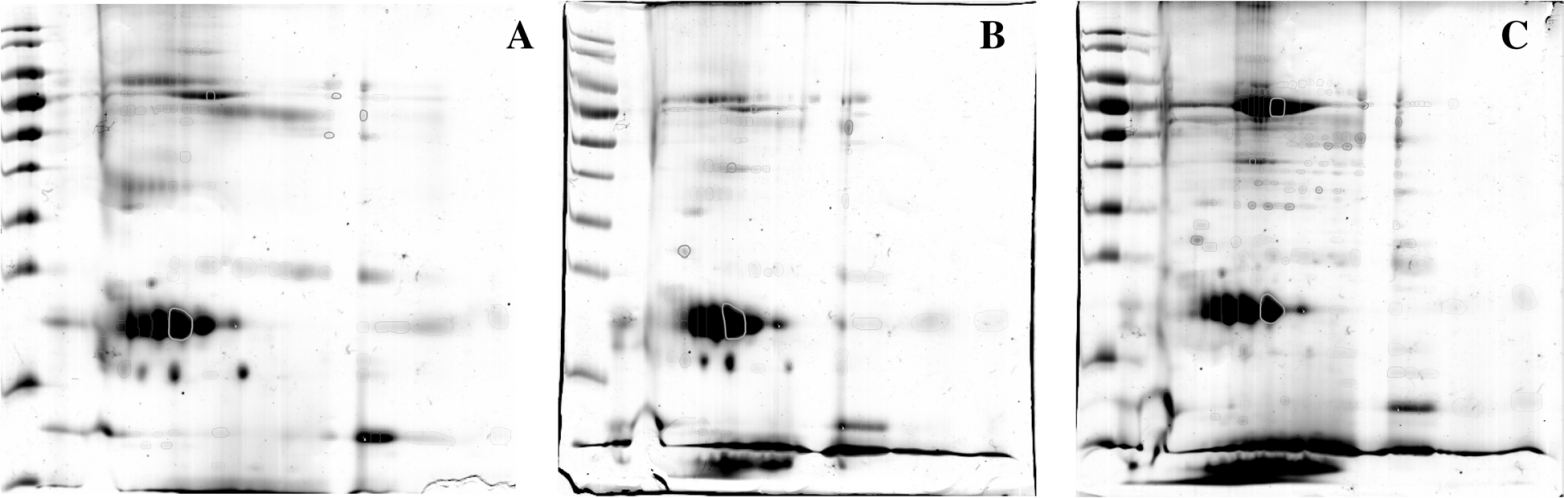
The 2-DE gel electrophoresis of tear protein collected with MT (A), ST (B), and OS (C). A seven-centimeter, pH 3–10 gradient strip was used. SDS-PAGE stained with Coomassie blue G-250. The gel is labeled with 14 candidate protein spots

The 2-DE results demonstrated a similar pattern of tear protein profiles in each tear collection method, although the intensities of the protein spots were different. The gel image program detected 249, 327, and 330 protein spots from MT, ST, and OS, respectively. Statistically significant differences among 14 protein spots were candidates for protein annotations. For determining the changes of protein spots among different tear collection methods, ST was used as the base line spot intensity. The 12 protein spots were upregulated and two protein spots were downregulated in OS compared to ST. For the MT collection method, one protein spot was upregulated and nine protein spots were downregulated when compared to ST (Fig. 2). The LC-MS/MS results (Table 1) demonstrated that 11 proteins were identified from 14 candidate protein spots (Fig. 3).

**Table 1:** Identification of candidate protein spots in healthy dogs’ tears using LC-MS/MS analysis

Spot ID	Identified proteins	Mass	pI	Protein score	Biological function
1	Actin, cytoplasmic 1	44,793	5.06	405	Retina homeostasis
2	Albumin	53,040	5.87	41	Antimicrobial
3	Albumin	53,040	5.87	40	Antimicrobial
4	Aldehyde dehydrogenase, dimeric NADP-preferring	51,193	6.44	58	Preventing corneal damage caused by ultraviolet light.
5	Alpha fetoprotein	73,962	5.95	1241	Heavy metal binding
6	Haptoglobin	36,890	5.72	208	Acute inflammatory response
7	Haptoglobin	36,890	5.72	124	Acute inflammatory response
8	Haptoglobin	36,890	5.72	61	Acute inflammatory response
9	Induced myeloid leukemia cell differentiation protein Mcl-1 homolog	36,884	5.49	22	Regulation of apoptotic process
10	Keratin 75	60,536	8.06	45	Establishment of skin barrier
11	Lactoferrin	70,444	8.00	47	Antimicrobial
12	Lipocalin 1	21,872	5.67	371	Antimicrobial
13	NCCRP1, F-box associated domain containing	38,977	5.97	31	Regulation of cell population proliferation
14	Stratifin	27,930	4.70	503	Establishment of skin barrier

The study demonstrated the downregulation of actin, cytoplasmic 1 (*P-value* = 0.005), albumin (*P-value* = 0.334), alpha fetoprotein (*P-value* = 0.634), haptoglobin (*P-value* = 0.334), induced myeloid leukemia cell differentiation protein Mcl-1 homolog (*P-value* = 0.334), keratin 75 (*P-value* = 0.00003), lactoferrin (*P-value* = 0.640), lipocalin 1 (*P-value* = 0.005), and stratifin (*P-value* = 0.024), when collecting the tear using MT compared to ST. When tears were collected using OS, proteins such as actin, cytoplasmic 1 (*P-value* = 0.006), albumin (*P-value* = 0.005), aldehyde dehydrogenase, dimeric NADP-preferring (*P-value* = 0.005), alpha fetoprotein (*P-value* = 0.000014), haptoglobin (*P-value* = 0.003), induced myeloid leukemia cell differentiation protein Mcl-1 homolog (*P-value* = 0.00004), lactoferrin (*P-value* = 0.022), NCCRP1, F-box associated domain containing (*P-value* = 0.013), and stratifin (*P-value* = 0.156), were upregulated.

**Fig. 4:**
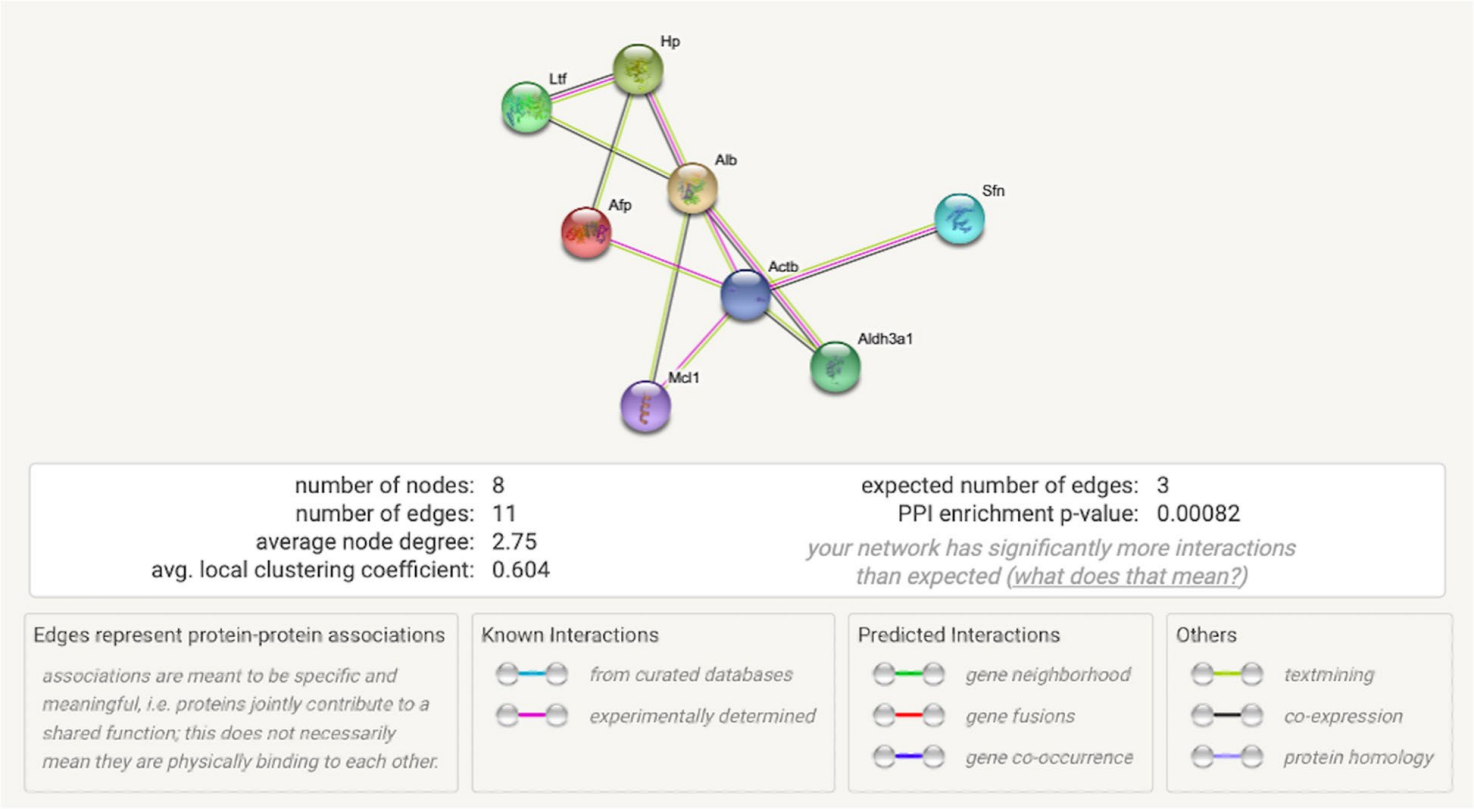
The pictures show protein-protein interactions and the statistical significance of the candidate proteins using STRING analysis. Alb = albumin, Hp = haptoglobin, Afp = alpha fetoprotein, Ltf = lactotransferrin, Actb = actin, cytoplasmic 1, Aldh3a1 = aldehyde dehydrogenase, dimeric NADP-preferring, Mcl1 = induced myeloid leukemia cell differentiation protein Mcl-1 homolog, and Sfn = stratifin

The possible interactions and line thickness in Fig. 4 refer to the strength of data support. The protein-protein interaction analysis can observe that proteins of albumin, haptoglobin, alpha fetoprotein, lactotransferrin, actin, cytoplasmic 1, aldehyde dehydrogenase, dimeric NADP-preferring, induced myeloid leukemia cell differentiation protein Mcl-1 homolog, and stratifin have strong interaction relationships.

## Discussion

Research for tear proteins in animals is valuable since it could lead to molecular biological investigation and the identification of a new biomarker for ocular and systemic diseases [6, 14, 23, 24]. Tear film is a body fluid widely used for proteomic studies because tears are non-invasively accessible. Due to the limited volume of tear film, pooled tear samples from several subjects can be used to overcome existing challenges [10, 16]. Nowadays, mass spectrometry allows for simultaneous accurate mass measures, in addition to the determination of the structural properties of molecules via tandem MS. Using chromatographic methods in conjunction with MS for protein identification provides the most complete view of a proteome distribution relative to isoelectric point (pI), molecular weight (MW), abundance, and interactions (i.e., protein–protein complex) [13]. Since the different tear collection methods affect proteomics in tear film, the results of proteomics studies using different tear collection methods are not directly comparable, and it is important to consider the potential impact of the collection method on protein concentration and expression [21, 25]. Green-Church (2008) examined the effect of the tear collection methods on the human tear proteome using a combination of one-dimensional (1-DE) and two-dimensional (2-DE) gel electrophoresis and mass spectrometry-based techniques. The result demonstrated that serum albumin expression from ST is greater than from MT [13]. Lamagna (2020) found the expression of aquaporin-1 from the OS collection was higher than from the ST in healthy dogs when using Western blot analysis [17]. Tear proteins have the potential as biomarkers for ocular and systemic diseases in humans and animals. For example, albumin and actin were used as biomarkers of cancers in dogs [12], and alpha fetoprotein was used as a tumor-specific biomarker (for example, in hepatocellular carcinoma) in humans [26], while lactoferrin and lysozyme were used as biomarkers of mucosal immune competence in humans [27], and lipocalin-1 was a candidate as a biomarker of dry eye syndrome [8], etc. Consequently, the findings of this study can be used to establish a proper tear collection method for any tear protein biomarkers in the future.

This was the first study to investigate and compare three different tear collection methods: microcapillary tube, Schirmer tear strip, and ophthalmic sponge. The findings of this study can be used to establish a proper tear collection method for future research on any interesting proteins. In this study, the ophthalmic sponges can absorb a larger volume of tears than other methods. Ophthalmic sponges exist in different material types, including polyvinyl alcohol and cellulose sponge materials [16]. Different material types display different properties of absorption. Polyvinyl alcohol sponge material (such as Merocel) with 100% open pores and no dead-end regions that retain residues is very absorbent and rapidly [28]. The natural cellulose sponge material, such as Weck-Cel sponges, is six times more absorbent than filter paper and maintains stiffness during the absorption process. Additionally, micropockets within Weck-Cel sponge materials could hold starch or sulfate residues [29]. Therefore, washing with varied solutions to eliminate the residues must occur when using these sponges. Some of the starch residue might be caught in the sponge’s polymer structure, where dissolving solutions cannot reach it. This makes removing the residues from the final sponge product difficult and may be utilized for cytokine separation from the sponge. Several cytokines and chemokines were found in tear samples collected with the Merocel sponge [28]. Additionally, the IgE value of tears recovered from capillaries was slightly lower than that of sponges. It is unclear whether these variations are due to differences in the adsorption of tear IgE or by evaporation from the larger surface area of the sponge during transportation [30]. Therefore, collecting tears with sponges that are greater in terms of absorption might reduce the fluid evaporation issue.

The tear samples from all dog groups that were collected with ST had the highest protein concentration, followed by the OS and MT. No statistically significant differences were found in each dog group and each of the tear collection methods. The low concentration of tear protein collected from MT is caused by small volume collection and less irritation to the ocular surface [16]. Additionally, while OS and ST were being used, some parts of this equipment were in contact with epithelial cells. Therefore, OS and ST retain ocular surface protein that may contaminate tear samples after the tear extraction process [1, 22, 31].

According to a recent study, the protein profiles from each tear collection method had a similar pattern but differed in terms of both the number and the intensity of the protein spots. Actin cytoplasmic 1, albumin, aldehyde dehydrogenase, dimeric NADP-preferring, alpha fetoprotein, haptoglobin, keratin 75, lactoferrin, lipocalin 1, and stratifin were among the 9 proteins previously found in the tear film [10, 20, 32]. We found some proteins that differed from different tear collection methods such as induced myeloid leukemia cell differentiation protein Mcl-1 homolog and NCCRP1, F-box associated domain containing NCCRP1, F-box associated domain containing.

Actin is the most abundant protein in the cytoplasm of animal cells. Its cellular functions range from organelle trafficking, regulation of cell shape, pathogen motility, cell migration, regulation of gene transcription, to the production of filaments that form cross-linked networks in the cytoplasm of cells [33–35]. Actin, cytoplasmic 1 was found in primary open angle glaucoma patients’ tears that could be involved in retina homeostasis [9]. Albumin has been detected in the tear film of humans [36], dogs [20], roadside hawks [37], and horses [38], etc. The main function of serum albumin is to bind to various substrates, including water, Ca^2+^, Na^+^, K^+^, fatty acids, hormones, bilirubin, and drugs. It can also limit the use of iron and the growth of bacteria [39]. Besides, inflammation of the ocular surface is associated with the presence of albumin in tear films. A previous study found that serum albumin in tear film increased significantly with each grade of conjunctivitis severity, with no differences between ST and MT [20]. De Freitas et al. (2008) found the increase in albumin levels in the tear film of dogs with cancer and identified them as the cancer biomarkers [12]. Furthermore, as previously described in dogs and other species, ocular disease disrupts the blood-tear barrier, allowing leakage of plasma compounds into the tear film. Aldehyde dehydrogenase, dimeric NADP-preferring (Aldh3a1) is a member of the ALDH superfamily of proteins that catalyze the NAD(P)-dependent oxidation of a wide range of endogenous and exogenous aldehydes [40]. Aldh3a1 constitutes the major fraction of the water-soluble protein in bovine and other mammalian corneas [41]. It has been reported to play a role in preventing ultraviolet light-induced corneal damage, which is consistent with the anti-apoptotic and cell growth-regulating roles of lens-crystallins [42]. The previous study revealed Aldh3a1 was significantly upregulated in pterygium and further increased in recurrent pterygium patients [40]. In addition, Aldh3a1 is highly expressed in non-small cell lung cancer (adenocarcinoma and squamous cell carcinoma) and in tobacco smokers versus nonsmokers [43]. Alpha fetoprotein is a serum glycoprotein with structural and physico-chemical properties similar to albumin. It is also present in small quantities in adults under normal conditions. Its biological role in embryonic and carcinogenesis remains obscure. The investigation of alpha fetoprotein for usage as a tumor-specific biomarker has been reported [44, 45], for example in hepatocellular carcinoma in humans [26]. Haptoglobin is an acute phase protein expressed through the activity of interleukin-6 [46]. A previous study found that haptoglobin in tear film increased with the severity of ocular lesions [10]. A higher serum haptoglobin level was found in calves infected with *Eimeria Zuernii* than in the control, which may be in response to an increased demand for haptoglobin in the repair process as a result of haemolysis. An inflammatory stimulus will upregulate the hepatic synthesis of haptoglobin [47]. Induced myeloid leukemia cell differentiation protein Mcl-1 homolog, also known as Bcl2-L-3, is encoded by *MCL1*. Bcl2-L-3 is an apoptosis-regulating protein in the B-cell lymphoma 2 family. It plays a key role in promoting cell survival [48–50]. The previous study demonstrated that Bcl2-L-3 prevented cell necrosis and has implications for the treatment of human hepatocellular carcinoma [51]. Keratin 75 is expressed primarily in hair follicles, nail beds, and lingual papillae, and was recently discovered in dental enamel. Keratins organize into heavily cross-linked networks of intermediate filaments, which provide mechanical rigidity to the cells and play important roles in cell-cell contacts as components of the desmosomal complexes [52]. The keratin proteins are usually present in the epidermal layer of skin and often not in the tear film in human. Therefore, the possible presence of keratin in tears could be caused by the habit of eye rubbing in people experiencing eye discomfort [53]. For tear lactoferrin, it was first reported by Masson in 1966 [54]. It is produced in the acinar cells of the lacrimal gland [55] and is present in the normal tears of mammals, including humans, cattle, bison, rabbits, dogs, cats, mice, koalas, and guinea pigs [56]. It has both anti-infective properties by suppressing bacterial growth by binding free iron, which is necessary for bacterial growth and preventing viral particles from entering cells, as well as anti-inflammatory properties by decreasing complement activation and scavenging free radicals [4]. Tear lipocalin-1 is one of the major tear proteins in humans [57]. Like lactoferrin, lipocalin is produced and secreted by the acinar cells [4]. It has many functions in tears, such as antimicrobial activity by binding to microbial siderophores, regulation of tear viscosity, anti-inflammatory activity, and endonuclease inactivation of viral DNA [3, 58]. Lipocalin-1 was a candidate as a biomarker of dry eye syndrome. Immune mediated dacryoadenitis is the most common etiology of dry eye syndrome. There is a progressive lymphocytic infiltration of the lacrimal gland that damages secretory tissues and decreases aqueous and tear protein (such as lipocalin-1) production [2, 16, 59]. NCCRP1, F-box associated domain containing is a type III membrane receptor protein that was isolated from the NCC of catfish and zebrafish. NCCRP-1 is a proline rich protein that has 2 glycosylation sites. Eighteen percent of the amino acids are serine, threonine, or tyrosine and function as potential phosphorylation sites [60]. NCCRP-1 plays a crucial role in the immune system by lysing tumor target cells, protozoan parasites, and virus-infected cells [61]. The 14–3-3 protein sigma, also known as stratifin, is particularly abundant in the stratified epithelium [62]. In addition, stratifin was reported in the tear film of humans and dogs. Stratifin has a crucial role in governing corneal epithelial cell differentiation and promotes cell cycle arrest when DNA is damaged [10, 63].

The current study demonstrated that albumin, Aldh3a1, alpha-fetoprotein, Bcl2-L-3, Hp, lactoferrin, and NCCRP1, F-box associated domain containing increase significantly in OS compared to ST and MT. Due to the size and hardness of the OS and ST, while using these methods, conjunctival injury may cause the serum proteins to leak into the tears through the blood-tear barrier [64]. Additionally, surface protein contamination could occur when using these methods [1, 22, 31]. Actin, cytoplasmic 1, keratin 75, lipocalin-1, and stratifin decrease significantly in MT compared to ST. Additionally, actin, cytoplasmic 1, and stratifin also decrease significantly in MT compared to OS. MT obtains a small volume of tear fluid and has an effect on the low concentration of tear components [16]. Thus, the protein concentration from MT could be lower than from other methods.

## Conclusion

The recent study supported that tear protein analysis is affected by different tear collection methods. The expression of tear protein in OS is higher than in other methods. Although ST is commonly used for clinical ocular examination and tear collection, it provides insufficient information to study particular tear proteins. From this research, we obtained knowledge of tear protein analysis using different tear collection methods, and this is the first step towards future studies about tear protein biomarkers of ophthalmic and systemic diseases in dogs.

## Methods

### Animals

The study was approved by the Ethics Committee on Animal Experimentation of Kasetsart University (ACKU64-VTN-008). Tear samples were collected from 16 healthy dogs of either gender and any breed. The age of the dogs ranged from 1 to 10 years old (mean ± SD = 5.06 ± 2.69 years) and the body weights were 3.1–26.4 kg (mean ± SD = 10.85 ± 6.41 kg). All dogs underwent physical and ocular examination by the veterinarian before tear collection. They were obliged to have normal physical examinations, including body temperature, heart rate, respiratory rate, pulse rate, capillary refill time, lung sounds, and lymph nodes palpation. Ocular examinations included ocular reflex (dazzle reflex, pupillary light reflex, and menace response), Schirmer tear test-1, measures of intraocular pressure, fluorescein staining, and portable slit lamp examination. The results of visual testing, ocular position, and all examinations were normal. The exclusion criteria included the presence of ocular or systemic diseases, recent ocular surgery, and receiving other than general maintenance medications. Informed consent was obtained from all owners before tear collection.

### Sample collection

Three different methods of tear collection were performed. No external stimulation and local anesthesia were required while collecting the tears from all tear collection methods. The MT was placed in contact with the inferior lacrimal lake until the tear was full in this microcapillary tube, in generally 5–10 minutes. The ST, made of Whatman paper no.4 (5-mm width × 35-mm length), was inserted into the ventrolateral conjunctival fornix until the wetness was 30 mm, in generally 1–3 minutes. The OS were modified from PVA ophthalmic surgical sponges. The sponge was inserted in the inferior lacrimal lake or beneath the lower eyelid for 1–3 minutes. The tear samples from each method were put into a sterile microtube and kept at − 80 °C until tear extraction. The dogs’ tears were obtained using a MT for the first method. ST and OS were performed for the second and third methods, respectively. Each method was carried out on a separate day.

### Tear protein extraction and concentration measurement

Each wetted ST and OS was transferred to 0.2 mL tubes, which were manually punctured at the bottom with an 18-gauge needle. Each tube was placed in a 1.5 mL microtube and centrifuged at 3884×g for 3 minutes at 4 °C. The tear samples were immediately collected at − 80 °C for further analysis. The tears were pooled from four dogs in each group. Each tear pool was incubated in an ultrasonic bath for 30 minutes at 4 °C. The protein concentration was measured using the Bradford assay at a 595 nm wavelength. Bovine serum albumin (BSA) was used as a standard solution.

### 2-DE

Amounts of 150 μg of pooled tear protein from each group were separated by isoelectric focusing (IEF) for the first dimension. Immobilized dry strips 7 cm in length, pH gradient 3–10 (Cytiva, USA) were used in this study. The first dimensional separation was performed by Ettan IPGphor II (GE Healthcare) using a focusing profile that increased the voltage to 12,000 Vhrs at 20 °C. Then the strips were equilibrated using an equilibration buffer containing 10 mg/mL dithiothreitol (DTT) for 30 minutes, followed by an equilibration buffer containing 25 mg/mL iodoacetamide (IAA) for 30 minutes. Then, 12.5% sodium dodecyl sulfate-polyacrylamide gel electrophoresis was performed on a mini VE vertical electrophoresis system (GE Healthcare) for the second dimension with a constant voltage of 140 V for 2 hrs 10 minutes at room temperature. The gels were stained with Coomassie blue G250 (CBG) with agitation overnight at room temperature whereupon several changes of MilliQ water were used to de-satin the gels. The gels were captured by ImageScanner II (GE Healthcare) and differential protein spots from triplicate gels were detected using Image Master 2-D Platinum version 7.0 (GE Healthcare). The different protein spots’ expression was compared by ANOVA. The 14 protein spots that showed statistically significant differences were submitted for protein identification using LC-MS/MS.

### In-gel digestion, LC-MS/MS analysis and database searching

The candidate protein spots were manually excised and then subjected to in-gel digestion and mass spectrometry. Protein spots were in-gel tryptic digested and dehydrated with 100% acetonitrile (ACN). Protein was digested with trypsin solution (10 ng/μL trypsin containing 50% ACN in 25 mM ammonium bicarbonate) for 18 hrs at 37 °C. Digested peptides were extracted with 0.1% formic acid (FA) in 50% ACN and lyophilized. The digested peptides were dissolved in 0.1% FA and then subjected to mass spectrometry analysis for protein identification. The LC-MS/MS systems were operated using Thermo Scientific Dionex Ultimate 3000 RSLCnano System with a captive spray ionization hybrid to Compact™ quadropole time-of-flight (Q-ToF) (Bruker Daltonik, Bremen, Germany). The LC separation was performed in a reversed-phase column of (Acclaim PepMap RSLC Column C18 NanoViper, 75 μm × 150 mm, particle size 2 μm) and protected by a guard column (C18 PepMap100, 300 μm × 5 mm, particle size 5 μm). The mobile phase composed of Solution A (0.1% FA in deionized water) and Solution B (80% ACN in deionized water). Elution of the peptides was separated at a flow rate of 0.3 μL/min under gradient conditions of 2 to 85% B for 50 minutes. The tandem mass spectrometry spectra were generated by Bruker qTOF Control Software. The files were converted to MGF files using Compass Data Analysis version 4.1 (Bruker Daltonik, Bremen, Germany) and searched using Mascot Server (Matrix Science, https://www.matrixscience.com) using the NCBInr database. The parameters for the Mascot search were peptide mass tolerance of 1 kDa; MS/MS ion mass tolerance of 1 Da; maximally one missed cleavage; and tryptic digestion. Only matched proteins with significance scores (*P-value* < 0.05) were reported.

### Protein-protein interaction network analysis

The protein-protein interaction analysis was performed using the STRING database version 11.5 (https://string-db.org). An interaction network analysis was created with proteins of the *Mus musculus* species to identify the proteins with a medium confidence score of 0.4 for interactions.

## Supplementary Information

**Additional file 1.**

## Data Availability

The datasets used and/or analyzed during the current study are available from the corresponding author on reasonable request.

## Abbreviations

2-DE: Two-dimensional electrophoresis
ACN: Acetonitrile
BSA: Bovine serum albumin
CBG: Coomassie blue G250
DTT: Dithiothreitol
FA: Formic acid
IAA: Iodoacetamide
IEF: Isoelectric focusing
LC-MS/MS: Liquid chromatography-tandem mass spectrometry
MT: Microcapillary tube
OS: Ophthalmic sponge
ST: Schirmer tear strip
