# Supplementary material for: Analysis and comparison of tear protein profiles in dogs using different tear collection methods
Source: BMC Vet Res. 2022 Dec 21;18:442. doi: 10.1186/s12917-022-03543-7 (PMC9768899; doi:10.1186/s12917-022-03543-7)
Supplement: Supplementary file 1 — Additional file 1. [file 12917_2022_3543_MOESM1_ESM.docx]

**Supplementary Information File 1**


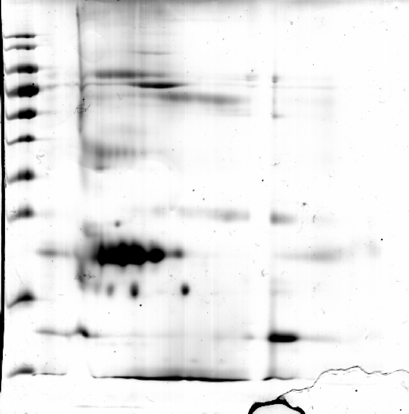

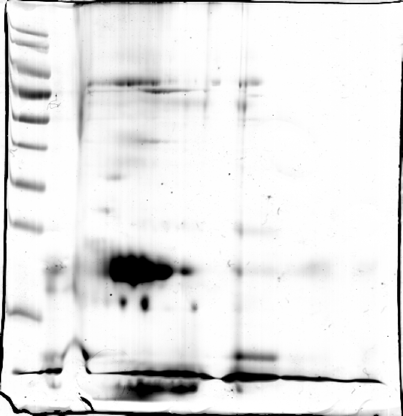

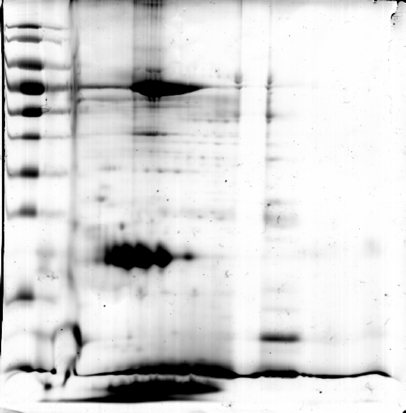


The full length original and uncropped gels of fig. 3 which show the 2-DE gel electrophoresis of tear protein collected with MT, ST, and OS, respectively. A seven-centimeter, pH 3-10 gradient strip was used. SDS-PAGE stained with Coomassie blue G-250.
